# Schwann Cell Transplantation Subdues the Pro-Inflammatory Innate Immune Cell Response after Spinal Cord Injury

**DOI:** 10.3390/ijms19092550

**Published:** 2018-08-28

**Authors:** Damien D. Pearse, Johana Bastidas, Sarah S. Izabel, Mousumi Ghosh

**Affiliations:** 1 The Miami Project to Cure Paralysis, University of Miami Miller School of Medicine, Miami, FL 33136, USA; dpearse@med.miami.edu (D.D.P.); jbastidas@gifbr.com (J.B.); izabelss@mymail.vcu.edu (S.S.I.); 2 The Department of Neurological Surgery, University of Miami Miller School of Medicine, Miami, FL 33136, USA; 3 The Neuroscience Program, University of Miami Miller School of Medicine, Miami, FL 33136, USA; 4 The Interdisciplinary Stem Cell Institute, University of Miami Miller School of Medicine, Miami, FL 33136, USA; 5 Department of Veterans Affairs, Veterans Affairs Medical Center, Miami, FL 33136, USA

**Keywords:** spinal cord injury, Schwann cell transplantation, inflammation, immunomodulation, MASCIS impactor, microglia, macrophage, inducible nitric oxide synthase, Arginase-1

## Abstract

The transplantation of Schwann cells (SCs) has been shown to provide tissue preservation and support axon growth and remyelination as well as improve functional recovery across a diverse range of experimental spinal cord injury (SCI) paradigms. The autologous use of SCs has progressed to Phase 1 SCI clinical trials in humans where their use has been shown to be both feasible and safe. The contribution of immune modulation to the protective and reparative actions of SCs within the injured spinal cord remains largely unknown. In the current investigation, the ability of SC transplants to alter the innate immune response after contusive SCI in the rat was examined. SCs were intraspinally transplanted into the lesion site at 1 week following a thoracic (T8) contusive SCI. Multicolor flow cytometry and immunohistochemical analysis of specific phenotypic markers of pro- and anti-inflammatory microglia and macrophages as well as cytokines at 1 week after SC transplantation was employed. The introduction of SCs significantly attenuated the numbers of cluster of differentiation molecule 11B (CD11b)^+^, cluster of differentiation molecule 68 (CD68)^+^, and ionized calcium-binding adapter molecule 1 (Iba1)^+^ immune cells within the lesion implant site, particularly those immunoreactive for the pro-inflammatory marker, inducible nitric oxide synthase (iNOS). Whereas numbers of anti-inflammatory CD68^+^ Arginase-1 (Arg1^+^) iNOS^−^ cells were not altered by SC transplantation, CD68^+^ cells of an intermediate, Arg1^+^ iNOS^+^ phenotype were increased by the introduction of SCs into the injured spinal cord. The morphology of Iba1^+^ immune cells was also markedly altered in the SC implant, being elongated and in alignment with SCs and in-growing axons versus their amoeboid form after SCI alone. Examination of pro-inflammatory cytokines, tumor necrosis factor-α (TNF-α) and interleukin-1β (IL-1β), and anti-inflammatory cytokines, interleukin-4 (IL-4) and interleukin-10 (IL-10), by multicolor flow cytometry analysis showed that their production in CD11b^+^ cells was unaltered by SC transplantation at 1 week post-transplantation. The ability of SCs to subdue the pro-inflammatory iNOS^+^ microglia and macrophage phenotype after intraspinal transplantation may provide an important contribution to the neuroprotective effects of SCs within the sub-acute SCI setting.

## 1. Introduction

Spinal cord injury (SCI) leads to local cell death, glial reactivity, and the disruption and demyelination of axonal tracts important for the conveyance of motor and sensory information [1]. These deficits are often pronounced and permanent due to the absence of restorative treatments clinically. Integral to the processes of secondary tissue damage and degeneration as well as angiogenesis and wound resolution is the innate immune response, comprising the activation of microglia and macrophages [2,3,4]. Immunomodulatory drug therapies that selectively inhibit or enhance components of the innate immune response have been shown to provide neuroprotection, facilitate repair, and enhance functional recovery [5,6,7,8,9]. The introduction of exogenous cells intravenously or intraspinally after SCI can also produce potent immunomodulatory effects that may be a key component of their reparative action. Transplanted bone marrow stromal and mesenchymal stem cells (BMSCs and MSCs) secrete growth factors and cytokines peripherally and locally that can alter the polarization of the immune response from one that is pro-inflammatory to one that is anti-inflammatory, enhancing tissue protection and reducing axonal degeneration after SCI [10,11,12]. Similarly, the intralesional delivery of neural precursor cells (NPCs) has been reported to inhibit microglia and macrophage activity, antagonize pro-inflammatory cytokine production, and reduce levels of inducible nitric oxide synthase (iNOS) [13,14,15].

In models of SCI, the intralesional transplantation of Schwann cells (SCs) has been shown to improve tissue preservation, axon growth, myelination, and functional recovery [16,17,18,19,20]. The interplay between Schwann cells (SCs) and cells of the innate immune system is essential to the intrinsic regenerative properties of peripheral nerves [21,22,23,24,25,26,27]. SCs produce chemokines and cytokines that are important for macrophage recruitment and myelin removal during the processes of Wallerian degeneration and ensuing axonal regeneration [24,28,29]. Our understanding of the influence of SCs on the innate immune system and the role of these interactions in mediating the protective and reparative actions of SCs after SCI remains limited. The current investigations sought to examine the immunomodulatory effects of SC transplantation in the sub-acute phase of SCI.

## 2. Results

### 2.1. Sub-Acute Transplantation of Schwann Cells Intraspinally Reduces Iba1 and CD68 Cells within the Lesion

**Figure 1:**
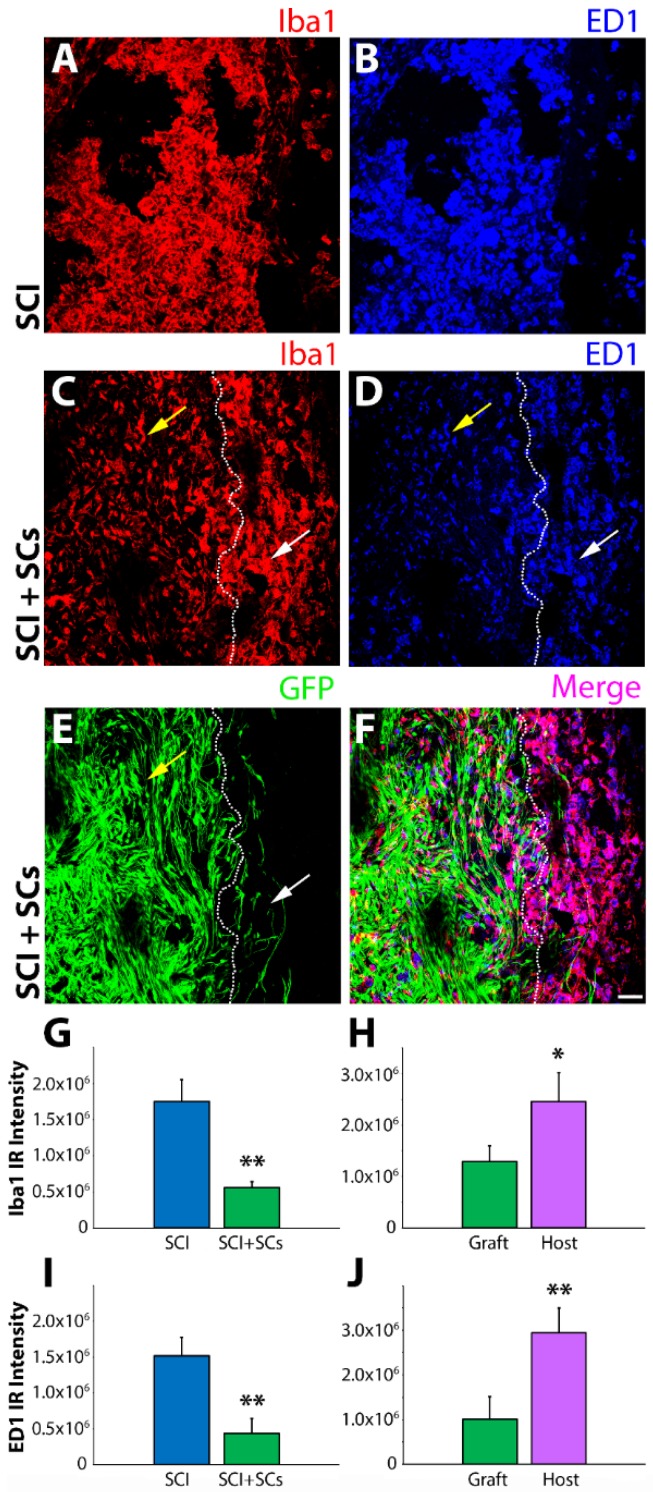
Immunoreactivity for ionized calcium-binding adapter molecule 1 (Iba1) and cluster of differentiation molecule 68 (CD68) was reduced within the lesion–Schwann cell (SC) implant of transplanted animals after spinal cord injury (SCI). Confocal micrographs of horizontal spinal cord sections from SCI control and SCI, enhanced green fluorescent protein (EGFP-SC)-transplanted animals (*n* = 4) at 2 weeks post-injury (1 week post-transplantation) immunostained for Iba1 (red) and CD68 (blue). In SCI control tissue, there was significant infiltration of both Iba1 and CD68 immune cells within the lesion (**A**,**B**). In contrast, in EGFP-SC-transplanted animals, the numbers of Iba1 and CD68 immune cells was greatly attenuated within the lesion–SC implant (**C**–**F**). Quantification of fluorescent intensity showed that EGFP-SC transplantation led to reductions in both Iba1 (**G**) and CD68 (**I**) that were more pronounced within the lesion than in adjacent host tissue (**H**,**J**). Results expressed as mean ± standard error of the mean (SEM). Statistical significance indicated at * *p* < 0.05 and ** *p* < 0.01 compared with SCI controls. Images were acquired at 20× objective magnification. Yellow arrows indicate the lesion-SC implant and white arrows the perilesional area. Scale bar = 50 μm.

At 7 days following transplantation into the lesion site, fluorescent intensity of innate immune cells markers within the lesion–SC implant were comparatively assessed between grafted and SCI-only controls. Injured spinal cord tissue sections were probed with a combination of Iba1, a calcium-binding protein and universal marker of both resting and activated forms of macrophages and microglia, as well as with CD68, a specific marker of activated phagocytes [30,31,32]. Fluorescent intensity measurements revealed a significant reduction in both Iba1 and CD68 within SC-transplanted SCI animals compared to SCI controls. The lesion and perilesional regions of the SCI-only controls were densely populated with Iba1 and CD68 macrophages and microglia (Figure 1A–F) compared with significantly fewer immune cells, particularly within the lesion–SC implant, of transplanted animals (Figure 1C,D). Perilesional areas of SC-transplanted animals showed similar Iba1 and CD68 fluorescent intensities to those same regions of SCI controls (Figure 1G–J).

### 2.2. SC Transplantation Alters Innate Immune Cell Phenotypes after SCI

**Figure 2:**
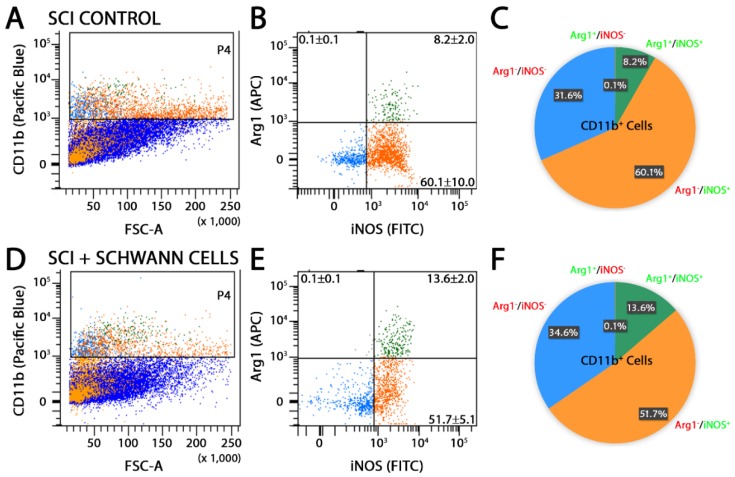
SC transplantation shifted the CD11b immune cell population from an Arg1^−^iNOS^+^ pro-inflammatory to an intermediate Arg1^+^iNOS^+^ phenotype after SCI. Representative images of flow cytometry analysis and pie charts of CD11b population dynamics at 14 days post-injury (7 days post-transplantation) show, compared with SCI controls (**A**–**C**), a decreased percentage of CD11b cells stained with Arg1^−^iNOS^+^ and an increased percentage for Arg1^+^iNOS^+^ in animals receiving SC transplants (**D**–**F**). Results are expressed as mean ± standard deviation (SD). Abbreviations on the graphs are: Fluorescein isothiocyanate (FITC), Allophycocyanin (APC) and Forward Scatter (FSC-A). For panels (**B**,**E**), the blue dots represent the CD11b population that is iNOS^−^-Arg1^−^, the orange dots represent the CD11b population that is iNOS^+^-Arg1^−^ and the green dots represent the CD11b population that is double positive for both iNOS^+^-Arg1^+^. These colored dots are also shown in the forward scatter plots of panels (**A**,**D**).

**Figure 3:**
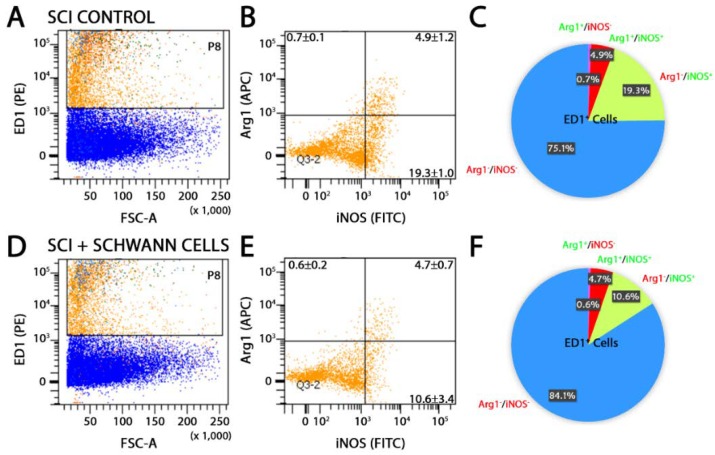
CD68 immune cells with a pro-inflammatory Arg1^−^iNOS^+^ phenotype after SCI are reduced by SC transplantation. Representative images of flow cytometry analysis and pie charts of CD68 population dynamics at 14 days post-injury (7 days post-transplantation) reveal that CD68 immune cells with a pro-inflammatory Arg1^−^iNOS^+^ phenotype after SCI (**A**–**C**) are reduced by the intraspinal transplantation of SCs (**D**–**F**). Results are expressed as mean ± SD. For panels (**A**,**D**), the orange dots represent the ED1 population that were gated based on their forward and side scatter from the total events (blue) that were acquired. The orange dots in the different quadrants of (**B**,**E**) represent the ED1 population that was positive for either Arg1 (top left quadrant) or iNOS (bottom right quadrant) or double positive for both markers (top right quadrant).

**Figure 4:**
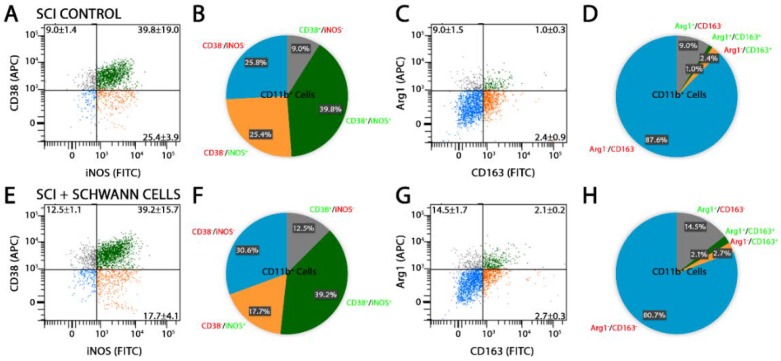
CD11b immune cells expressing a highly pro-inflammatory CD38^+^iNOS^+^ phenotype or anti-inflammatory Arg1^+^CD163^+^ form after SCI were unaltered by SC transplantation. Representative images of flow cytometry analysis and pie charts of CD11b population dynamics at 14 days post-injury (7 days post-transplantation) reveal that CD11b immune cells with a highly pro-inflammatory CD38^+^iNOS^+^ phenotype after SCI (**A**,**B**) are not altered by SC transplantation (**E**,**F**). Similarly, CD11b immune cells with a highly anti-inflammatory Arg1^+^CD163^+^ phenotype were unchanged across SCI controls (**C**,**D**) and SC-transplanted groups (**G**,**H**). Results are expressed as mean ± SD. For panels (**A**,**B**), the blue dots represent the CD11b population that were CD38^−^iNOS^−^, the orange dots represent the CD11b population that were CD38^−^iNOS^+^, the gray dots represent the CD11b population that were CD38^+^iNOS^−^ and the green dots represent the CD11b population that were double positive for both CD38^+^iNOS^+^. For panels (**C**,**D**), the configuration for the colored dots is the same for the representation labeling, single, double or absent, though the proteins Arg1 and CD163 are represented rather than CD38 and iNOS.

**Figure 5:**
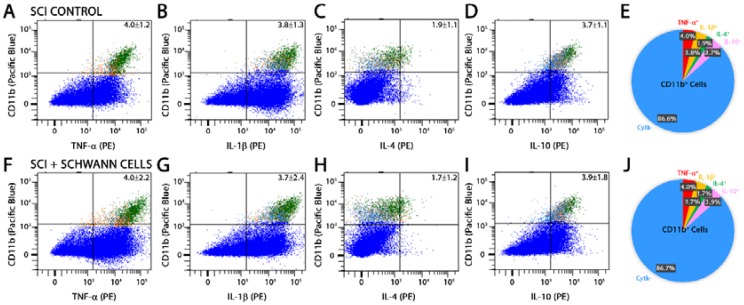
Cytokine profile of CD11b^+^ cells is unaltered by SC implants after SCI. Representative images of flow cytometry analysis and pie charts of CD11b population dynamics at 14 days post-injury (7 days post-transplantation) show that CD11b immune cells have unaltered expression of pro- (TNF-α, IL-1β) and anti-inflammatory (IL-4, IL-10) cytokines comparatively among SCI controls (**A**–**E**) and SC-transplanted groups (**F**–**J**). Results are expressed as mean ± SD. For panels (**A**–**D**,**F**–**I**) the blue dots represent the cell population that were CD11b^−^ or did not express the selected cytokines, whereas the green dots represent the CD11b^+^ population that expressed CD11b and the cytokine of interest (top right quadrant).

Flow cytometry analysis of the injured spinal cord segment was performed at 14 days after injury in SC-transplanted and SCI control animals using CD11b or CD68, in combination with antibodies towards either pro-inflammatory molecules, iNOS [5,33] and cluster of differentiation molecule 38 (CD38) [34,35], or anti-inflammatory markers, arginase-1 (Arg1) and cluster of differentiation molecule 163 (CD163) [33]. The production of pro-inflammatory cytokines, tumor necrosis factor-α (TNF-α) and interleukin-1β (IL-1β), and anti-inflammatory cytokines, interleukin-4 (IL-4) and interleukin-10 (IL-10), was also probed. SC transplantation significantly reduced the percentage of CD11b^+^Arg1^−^iNOS^+^ pro-inflammatory cells from 60.1 to 51.7% while enhancing the number of CD11b^+^Arg1^+^iNOS^+^ cells, an intermediate phenotype, from 8.2 to 13.6% (Figure 2). Numbers of CD11b^+^Arg1^+^iNOS^−^ anti-inflammatory cells were unaffected by SC transplantation compared with SCI controls. These findings were corroborated by a similar reduction in CD68^+^Arg1^−^iNOS^+^ pro-inflammatory cells from 19.3 to 10.6% following SC transplantation (Figure 3). Another pro-inflammatory immune cell marker, CD38, was largely unchanged in CD11b cells after SC transplantation (Figure 4A,B,E,F). Analysis of CD11b immune cells that were CD163^+^, a scavenger receptor associated with anti-inflammatory activities, showed that there was no change with SC transplantation after SCI (Figure 4C,D,G,H), though numbers of cells expressing both Arg1 and CD163 were reduced 9.0 to 14.5% following SC transplantation. Comparison of pro- and anti-inflammatory cytokine production in CD11b cells by flow cytometry showed no significant differences between SC-transplanted and SCI controls (Figure 5).

**Figure 6:**
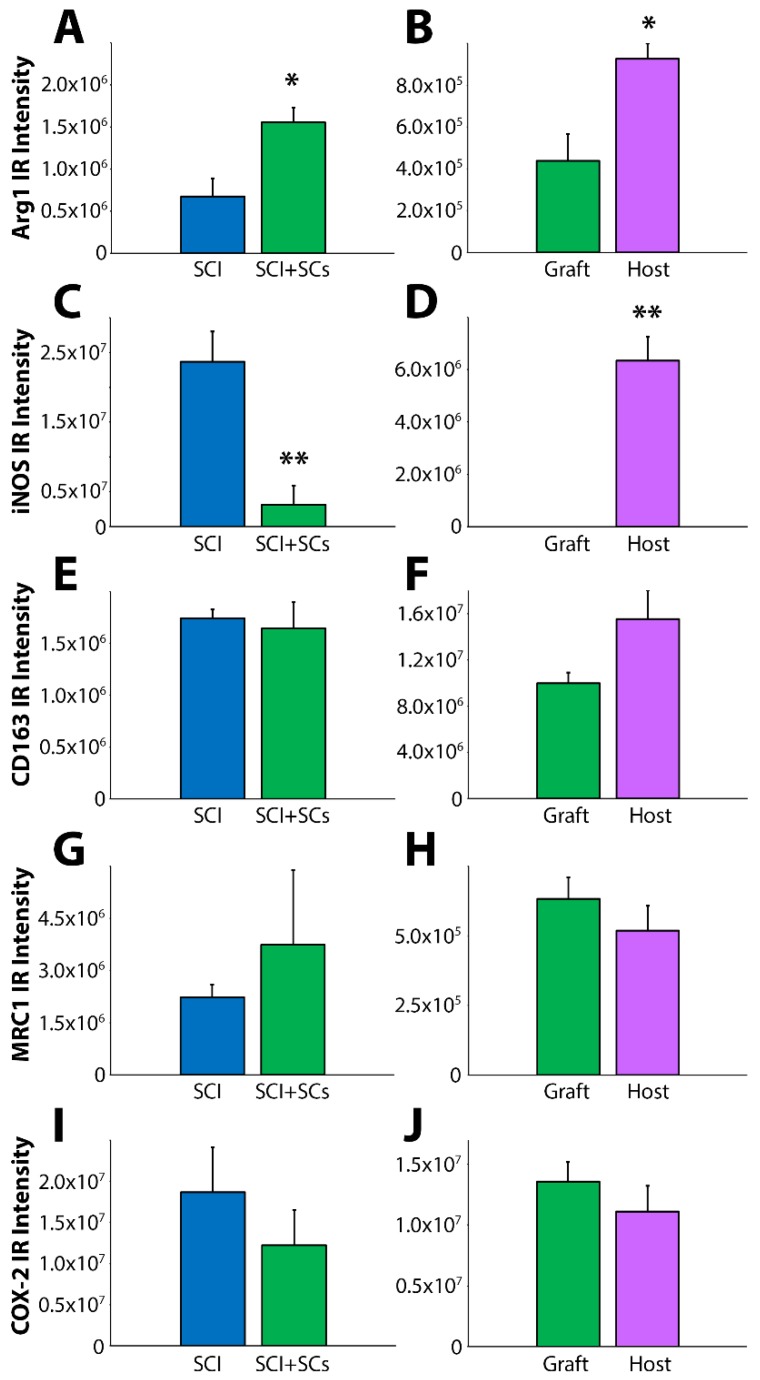
Immunohistochemistry revealed a significant pro- to anti-inflammatory switch in staining from iNOS to Arg1 within the injured spinal cord of SC-transplanted animals. The presence of pro- and anti-inflammatory markers of immune cell phenotype was measured quantitatively within the lesion and adjacent host spinal tissue by immunohistochemistry with specific fluorescent-conjugated antibodies and subsequent measurement of fluorescent intensity. Arg1 immunoreactive intensity was significantly increased following EGFP-SC transplantation (**A**) and this enhancement was most evident in the adjacent host tissue (**B**). In contrast, iNOS immunoreactivity was dramatically attenuated following EGFP-SC transplantation (**C**) and was virtually absent from the lesion–SC implant (**D**). EGFP-SC transplantation was not accompanied by changes in immunoreactivity for the others markers examined; CD163 (**E**,**F**), mannose receptor C-type 1 (MRC1; (**G**,**H**)) and cyclooxygenase-2 (COX-2; (**I**,**J**)). Results expressed as mean ± SEM. Statistical significance indicated at * *p* < 0.05 and ** *p* < 0.01 compared with SCI controls.

**Figure 7:**
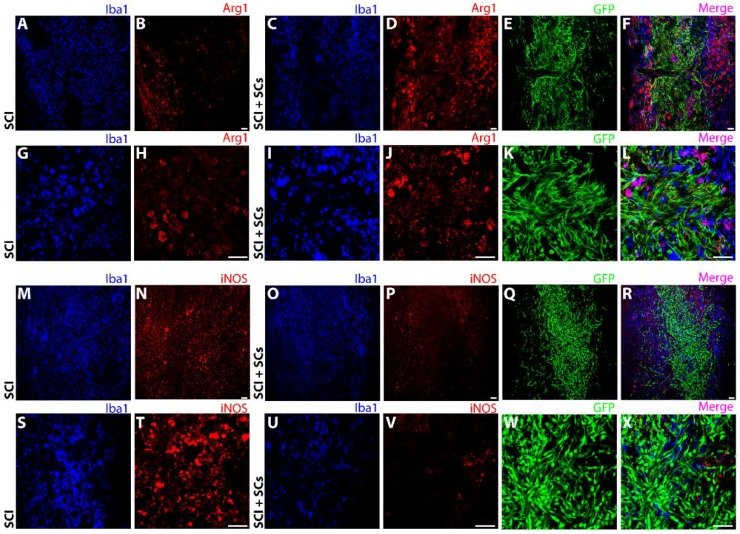
Lesional and perilesional changes in Arg1 and iNOS were observed after SCI and SC transplantation. Immunohistochemical staining showed a pronounced increase in Arg1 immunoreactivity (red) from SCI controls (**A**,**B**,**G**,**H**) to EGFP-SC-transplanted (green) animals (**C**,**D**,**I**,**J**). The increase in Arg1 was most pronounced in the perilesional region and adjacent host spinal cord tissues (**E**,**F**,**K**,**L**). In contrast, compared with SCI controls (**M**,**N**,**S**,**T**), iNOS immunoreactivity (red) was dramatically reduced after EGFP-SC transplantation (**O**,**P**,**U**,**V**), being virtually absent from the lesion–SC implant (**Q**,**R**,**W**,**X**). Total innate immune cells identified using Iba1 (blue). Triple staining of EGFP, Iba1 and either Arg1 or iNOS are shown in the merged images (pink; (**F**,**L**,**R**,**X**)). Images were acquired at 10× (**A**–**F**, **M**–**R**) and 40× (**G**–**L**, **S**–**X**) objective magnifications. Scale bar = 50 μm.

**Figure 8:**
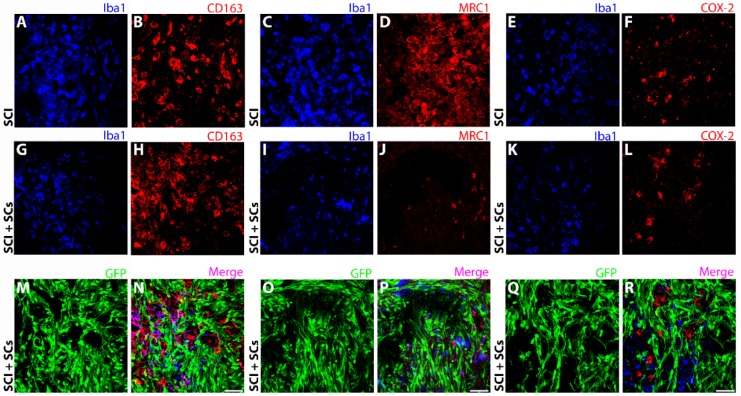
CD163, MRC1, and COX-2 immunoreactivity was unchanged with SC transplantation though lesional and perilesional disparities were observed. Immunoreactivity for CD163 (red) was similar among SCI control (**A**,**B**) and EGFP-SC-transplanted (green) (**G**,**H**,**M**,**N**) animals, while MRC1 (red) did show a reduction in immunoreactivity from the SCI control (**C**,**D**) within the lesion–SC implant (**I**,**J**,**O**,**P**) but not overall. No change in the immunoreactivity for COX-2 (red) was seen between SCI control (**E**,**F**) and EGFP-SC transplant groups (**K**,**L**,**Q**,**R**). Total innate immune cells identified using Iba1 (blue). Triple staining of EGFP, Iba1 and CD163, MRC1 or COX-2 are shown in the merged images (pink; (**N**,**P**,**R**)). Iba1 Images were acquired at 40× objective magnification. Scale bar = 50 μm.

In parallel to flow cytometry, immunohistochemical staining of spinal tissues from another cohort of animals from each group was employed. A 3-fold increase in Arg1 immunoreactivity was observed in Iba1 cells in SC-transplanted animals (Figure 6A) that was largely restricted to changes within the lesion–SC implant area rather than the perilesional region (Figure 6B and Figure 7A–L). Conversely, iNOS immunoreactivity was dramatically reduced in Iba1 cells in SC-transplanted animals (Figure 6C). Again, this change was largely restricted to the lesion–SC implant as robust iNOS immunoreactivity remained in the perilesional region in transplanted animals (Figure 6C,D and Figure 7M–X). Changes in the immunoreactivity of other pro- and anti-inflammatory markers were not observed; CD163 (Figure 6E,F and Figure 8A,B,G,H,M,N), mannose receptor C-type 1 (MRC1) (Figure 6E,F and Figure 8C,D,I,J,O,P), and cyclooxygenase-2 (COX-2) (Figure 6E,F and Figure 8E,F,K,L,Q,R).

### 2.3. Macrophages and Microglia within SC Transplants Exhibited a Dramatic Change in Cell Morphology and Were Found in a Parallel Association with SCs and Axons

**Figure 9:**
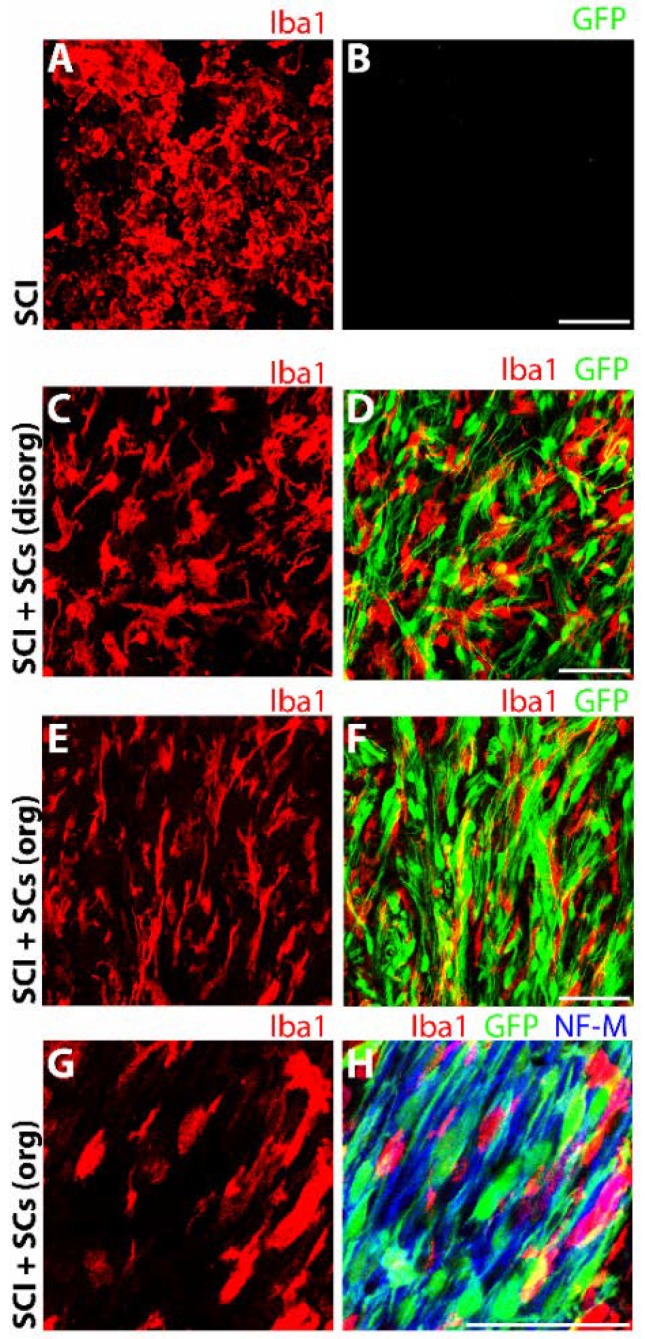
Pronounced morphological changes occur in Iba1 (red) microglia and macrophages after SCI in the presence of transplanted SCs (green). Microglia and macrophages immunoreactive for Iba1 exhibited a rounded, amoeboid morphology in the lesion and perilesional regions of SCI controls (**A**,**B**). In contrast, in EGFP-SC-transplanted animals, Iba1 immune cells displayed either a ramified, multipolar phenotype (**C**,**D**), commonly associated with resting or surveillant cells, or an elongated, rod-shaped form that aligned in parallel to that of SCs and axons (blue) (**E**–**H**). Images were acquired at 60× objective magnification for (**A**–**F**) and at 140× for (**G**,**H**). Scale bar = 50 μm.

Macrophages and microglia displayed distinct morphological characteristics within the lesion–SC implant compared with those observed either in SCI controls or in the adjacent host spinal tissue of grafted animals. Examination of Iba1 cells in SCI controls revealed a morphology consisting of a rounded, amoeboid form with short processes that is characteristic of an activated phagocyte (Figure 9A). In contrast, within the lesion–SC implant of transplanted animals, Iba1 cells exhibited two different and distinct morphologies that appeared to mirror the morphology of the SCs within that region. In the periphery of the SC graft, where the SCs were in low density and stellate in appearance, Iba1 cells exhibited a ramified morphology, with a large cell body and long, highly branched processes that is often associated with the homeostatic or surveillant phenotype of microglia (Figure 9G,H). In contrast, within the main body of the SC implant, where the SCs were densely packed, elongated and lined up in parallel with ingrowing axons, the Iba1 cells adopted a similar morphology to the SCs, with a rod-like shape and parallel positioning to the SCs (Figure 9E,F) and neurofilament-positive axons (Figure 9G,H).

## 3. Discussion

The current investigation was designed to evaluate the effects of Schwann cell transplantation on cellular responses of the innate immune system following SCI. The interaction of SCs with cells of the innate immune response has been shown to be essential for peripheral nerve repair [21,24,27,29], though our understanding of the effects of transplanted SCs on macrophages and microglia within the injury milieu following SCI remains limited. The transplantation of SCs into the sub-acutely injured spinal cord at one week was associated with a marked decrease in Iba1^+^ and CD68^+^ immune cells, particularly within the lesion–transplant site. Phenotypic changes in CD11b^+^ and CD68^+^ cells in response to SCs were observed including a dramatic reduction in pro-inflammatory iNOS^+^Arg^−^ cells and an increase in an intermediate Arg^+^iNOS^+^ phenotype that would be indicative of a change in functional state towards the suppression of inflammation [5]. The morphology of Iba1^+^ immune cells were also changed within the SC transplant from a rounded, amoeboid form to an elongated, spindle shape that is often associated with a reparative function [36,37]. Flow cytometry analysis of CD11b^+^ cells revealed no changes in their immunoreactivity for pro- (TNF-α, IL-1β) and anti-inflammatory (IL-4, IL-10) cytokines. The ability of SCs to subdue the pro-inflammatory iNOS^+^ microglia and macrophage phenotype after intraspinal transplantation may play an important contribution to the neuroprotective effects of SCs within the sub-acute SCI setting and enable a more expedient switch in the innate immune response towards one conducive to repair. 

The phenotype and function of microglia and macrophages following SCI has been described in terms of a dichotomy of pro- and anti-inflammatory forms [5,38,39]. An injury-induced bias towards the pro-inflammatory phenotype has been reported as playing a causative role in the deleterious actions of the innate immune system towards secondary tissue damage, axon degeneration and demyelination, as well as abortive axon regeneration and repair [40]. The immune response is driven by lesion-associated factors and these pro-inflammatory macrophages and microglia appear to persist within the chronic SCI lesion throughout life [41,42]. Conversely, the therapeutic use of pharmaceuticals, cells, and biomaterials that alter the injury milieu and switch the balance of the innate immune response towards an anti-inflammatory state has been associated with neuroprotection, repair, and improved functional recovery [5,7,9,11,42]. Acute transplantation of MSCs, intravenously [43,44] or intraspinally [12], has been shown to provide neuroprotection after SCI and have peripheral or local effects, respectively, on the innate immune system. Immunomodulation by MSCs involves a phenotypic conversion of macrophages [45] from a pro-inflammatory iNOS^+^CD16^+^ phenotype to one that is anti-inflammatory, Arg1^+^CD206^+^, an action mediated by growth factors and cytokines including tumor necrosis factor a-induced protein 6 (TGS6) [46] and tissue inhibitor of metalloproteinases-3 (TIMP-3) [47]. The phenotypic conversion of macrophages is accompanied by reduced secretion of TNF-α and interferon gamma (IFN-γ) and increased IL-4 and IL-10 that diminish the cytotoxicity of the lesion environment [12]. Similarly, the intraspinal transplantation of neural progenitor cells has been demonstrated to suppress the pro-inflammatory iNOS immune cell phenotype after SCI to mediate their beneficial reparative effects [14].

SCs are key orchestrators of successful regeneration following peripheral nerve injury [27,48,49]. Acting in concert with an intrinsic neuronal regenerative potential, favorable innate immune response, and growth-supportive nerve extracellular matrix, SCs produce factors that alter the injured nerve environment [50], recruit macrophages [28], promote myelin removal [51], and stimulate axon growth [48] towards the restoration of nerve conduction and function. Despite the importance of the interplay of SCs and macrophages in nerve repair, our understanding of the effects of SCs on cells of the innate immune system after transplantation into the injured spinal cord remains limited. Prior work has shown that when SCs are combined with olfactory ensheathing cells (OECs) for transplantation after SCI, which enhances their reparative efficacy [52], there is a switch in microglia and macrophage phenotype, from iNOS^+^CD16/32^+^ to Arg1^+^CD206^+^, as well as a reduction in levels of TNF-α and IL-6 and increased IL-10 and IL-13 [53]. Here we show that transplanted SCs alone are capable of inducing a transformation of Iba1, CD11b, or CD68 immune cells from a pro- to an anti-inflammatory phenotype, though changes in cytokine production were not observed. The disparity in cytokine changes between studies may therefore be related to the effects of OEC co-transplantation or differences in how cytokine measurement was performed—whole tissue homogenates in the former study versus the specific measurement of cytokine production in CD11b^+^ immune cells by flow cytometry in the current work. Though changes in the phenotype of innate immune cells after SCI with SC transplantation could be the result of secreted or cell contact factors that could alter the expression of pro- and anti-inflammatory molecules within the immune cells to switch their phenotype and function, these changes could also have occurred as a result of an alteration in the infiltration or drainage from the lesion of specific innate immune cell populations in response to SC transplantation. Pre-labeling and tracking experiments of selected immune cell populations (pro- or anti-inflammatory) may allow further elucidation of the mechanism by which SC transplantation alters cell phenotype after SCI. In the injured peripheral nervous system (PNS), SCs express toll-like receptors (TLRs) that are stimulated and up-regulated acutely after injury, enabling SCs to play a sentinel role through their expression of chemokines including TNF-α, iNOS, and MCP-1, which recruit resident and hematogenous macrophages [23]. Macrophage recruitment permits Wallerian degeneration and myelin clearance in the distal nerve stump where SCs secrete trophic factors and extracellular matrix molecules to support neuron survival, growth, and remyelination [50]. The marked change in Iba1^+^ microglia and macrophage morphology when in the presence of SCs and ingrowing axons after SCI would imply that SCs significantly affected their function. Whereas rounded, amoeboid cells are highly activated and immunoinflammatory [54], rod-shaped microglia and macrophages are usually present during tissue regeneration and repair [37]. Additionally, SC transplantation was associated with an overall reduction in CD68 immunoreactivity. CD68 or macrosialin (clone ID ED1), is a protein associated with the lysosomal membrane that has been previously detected at high levels in innate immune cells that are activated phagocytes, but has limited expression in tissue resident macrophages and microglia [30,31,32]. A reduction in CD68^+^ innate immune cells with SC transplantation may therefore be indicative of a suppression of immune cell activation and/or their conversion to a phagocytic form. The examination of this change at a single time point after SCI and SC transplantation, however, makes it difficult to determine whether SC transplantation induced a suppression of the phagocytic state or hastened a resolution of this activity, as observed in PNS injury repair [28,50].

In the process of PNS regeneration a temporal switch in the innate immune system occurs in the T helper cell subsets (Th) 1 and 2 from a Th1 to Th2 response that is critical for angiogenesis and reparative matrix deposition [24]. Therapies that hasten the switch of the immune response from Th1 to Th2 have been shown to also enhance the speed of recovery after PNS injury [23,24,49]. In the CNS, transplanted SCs are restricted to the lesion site by the glial scar and its inhibitory matrix components [55], such as chondroitin sulfate proteoglycans (CSPGs), preventing both SC migration to regions of Wallerian degeneration, to influence immune cell responses directly, as well as the ability of SCs to guide supraspinal axons into and from the lesion. Therefore, the capacity of SCs to stimulate the conversion of the innate immune response from Th1 to Th2 after SCI in the present study, particularly in the adjacent host spinal cord tissue, may have been limited by the inability of SCs to leave the lesion site without additional adjunct approaches targeting their migration [55].

In conclusion, SC transplantation into the sub-acutely injured spinal cord produces a change in the innate immune system from that of a Th1 to a Th2 response that does not normally occur after SCI but is known to be an important process in PNS repair. This transition in the innate immune response, influenced by extracellular matrix (ECM) and growth factors produced by SCs, may be a critical component of the ability of SCs to promote protection, repair, and functional recovery when transplanted after SCI.

## 4. Materials and Methods

### 4.1. Reagents

Antibodies used were rabbit anti-Iba1 polyclonal antibody (catalog # 019-19741; FUJIFILM Wako Chemicals U.S.A Corporation, Richmond, VA, USA), chicken polyclonal to Iba1 (catalog # ab139590; Abcam, Cambridge, MA, USA), chicken anti-GFP antibody (catalog # AB16901; EMD Millipore-Sigma, St. Louis, MO, USA), mouse CD68/clone CD68 (catalog # MCA341R; BIO-RAD, Hercules, CA, USA), chicken anti-green fluorescent protein (catalog # AB16901; Millipore-Sigma), rabbit Arginase-1 (catalog # GTX109242; Gen eTex), rabbit anti-CD163 antibody (catalog # ab87099; Abcam), rabbit anti-MRC1 antibody (catalog # HPA045134; Millipore-Sigma), rabbit anti-COX2/Cyclooxygenase 2 antibody (catalog # ab15191; Abcam), rabbit polyclonal Neurofilament NF-M, (catalog # RPCA-NF-M; Encore Biotechnology, Gainesville, FL, USA), mouse anti-rat Cd11b:Pacific Blue (catalog # MCA275PB; BIO-RAD), human/mouse Arginase-1/ARG1 APC-conjugated (catalog# IC5868A; R&D systems, Minneapolis, MN, USA), NOS2 Alexa Fluoro 488 (catalog# 610331; BD Bioscience, San Jose, CA, USA), anti-rat CD68:RPE (catalog # MCA341PE; BIO-RAD), CD38 monoclonal antibody (14.27), eFluor 660, (catalog # 50-0380-80; ThermoFisher Scientific, Waltham, MA, USA), anti-rat CD163:FITC (catalog # MCA342F; BIO-RAD), PE anti-mouse/rat TNF alpha (catalog # 506104; Biolegend, San Diego, CA, USA), IL-1β antibody (E7-2-hILβ) PE (catalog # sc-32294 PE; Santa Cruz, Dallas, TX, USA), BD Pharmingen™ PE mouse anti-rat IL-4 (catalog # 555082; BD Bioscience), PE mouse anti-rat IL-10 Clone A5-4 (catalog # 555088; BD Bioscience), IL12, PE rat anti-mouse (catalog # 562038; BD Bioscience), PE anti-mouse CD206 (MMR), (catalog # 141705, Biolegend), and purified mouse anti-rat CD32 clone D34-485 (catalog # 55027; BD Pharmingen, San Jose, CA, USA). Secondary antibodies included goat anti-chicken IgG Alexa Fluor 488 (catalog #A-11039), goat anti-rabbit IgG Alexa Fluor 594 (catalog #A-11012), Goat anti-Mouse IgG Alexa Fluor 594 (catalog #R37121), goat anti-mouse IgG Alexa Fluor 405 (catalog #A-31553), goat anti-rabbit IgG Alexa Fluor 405 (catalog #A-31556), goat anti-rabbit IgG Alexa Fluor 647 (catalog # A-21245), and goat anti-mouse IgG Alexa Fluor 660 (catalog # A-21235), and were all from ThermoFisher Scientific (Waltham, MA, USA).

### 4.2. Schwann Cell Culture

Purified SCs were harvested from dissociated sciatic nerves of adult female Fischer rats as previously described [55,56]. Briefly, SCs were plated on dishes coated with poly-lysine and maintained in growth media composed of D10 (Dulbecco’s Modified Eagle’s Media (DMEM) + 10% Fetal Bovine Serum (FBS) and Pen-Strep) with three mitogens (D10+3M; 2 μM forskolin, 20 μg/mL bovine pituitary extract, and 10 ng/mL Heregulin). SCs were further passaged thrice in D10+3M followed with cryopreservation as stocks before experimental use. Prior to implantation into the injured spinal cord, SCs were thawed and grown to 80–85% confluency and transplanted. Unlabeled SCs were transplanted in only those animals used for flow cytometry analysis. SC purity was >95% as assessed by S100 immunoreactivity.

### 4.3. Introduction of Lentiviral Vectors into SCs

For in vitro infection of SCs with lentiviral vectors (LVs) encoding enhanced green fluorescent protein (EGFP), Passage 2 SCs at 45–50% confluence were transduced at a multiplicity of infection (MOI) of 50 overnight. Medium was refreshed the following day and cultures were maintained until Passage 4. A MOI of 50 produced an infection rate of >95% as determined by the presence of EGFP with an absence of toxicity. At Passage 4, SCs expressing EGFP were cryopreserved until experimental use where they were thawed, grown to 80–85% confluence, and transplanted. EGFP SCs were transplanted in only those animals used for immunohistological analysis.

### 4.4. Animals

Adult female Fischer rats (Harlan Company, *n* = 20; ~180–200 g) were housed according to NIH and The Guide for the Care and Use of Animals. All animal procedures employed in this study were approved by the Institutional Animal Care and Use Committee (IACUC) of the University of Miami (IACUC approval # 17-010, dated: 23 January 2017). 

### 4.5. Pre-Operative Preparation

Prior to surgical procedures, the animals were weighed and anesthetized with a mixture of 2% isoflurane and 30% oxygen. An adequate level of anesthesia was determined by monitoring the corneal and hindlimb withdrawal reflexes. The backs were shaved and aseptically prepared with chlorhexidine (Phoenix Pharmaceutical Inc., St. Joseph, MO, USA). Lacrilube ophthalmic ointment (Allergan Pharmaceuticals, Irvine, CA, USA) was applied to the eyes to prevent drying. Throughout the surgery, the rats were kept on a homeothermic blanket system (Harvard Apparatus Ltd., Kent, UK) to maintain body temperature at 37 ± 0.5 °C, as assessed by a rectal probe.

### 4.6. Moderate Thoracic Spinal Cord Contusion Injury

Spinal cord contusion was induced by The Multicenter Animal Spinal Cord Injury Study (MASCIS) impactor developed at New York University [57]. Briefly, a laminectomy was performed at thoracic vertebra level T8 to expose the dorsal surface of the spinal cord without disrupting the dura. The exposed spinal cord was moderately injured by dropping a 10.0 g rod from a height of 25.0 mm. The injury parameters (contusion impact height, velocity, and compression) were monitored. Animals were excluded immediately when height or velocity errors exceeded 7% or if the compression distance was not within the range of 1.75 to 2.25 mm. After injury, the muscles were sutured in layers and the skin closed with metal wound clips. The rats were allowed to recover in a warmed cage with water and food easily accessible. Post-operative care, including the administration of antibiotics, analgesics, and rehydrating fluids, was performed as described elsewhere [55]. At 1 week post-injury, the animals were randomly assigned into one of 2 groups; (i) injury only (*n* = 10); or (ii) injured with the transplantation of unlabeled SCs for flow cytometry experiments (*n* = 5 per group) or injured with EGFP-SC transplantation (*n* = 5 per group) for immunohistological assessments.

### 4.7. Intraspinal Implantation of Schwann Cells

Prior to intraspinal transplantation at the injury epicenter, either unlabeled SCs or EGFP-SCs in culture were harvested via trypsinization as described previously [58]. Unlabeled SCs were implanted in SCI animals that were subjected to flow cytometry analysis while EGFP-SC transplantation was employed in SCI rats that were subjected to immunohistological assessment of the immune cell phenotypes. Prior to implantation, unlabeled or EGFP-SCs were counted and suspended in DMEM-F12 medium. Cells were suspended to a final concentration of 3.3 × 10^5^ cells/μL and were kept on ice (<2 h) until transplantation. Animals were anesthetized and the original site of laminectomy was re-exposed. For animals in the SC group, a total of 2.0 × 10^6^ unlabeled SCs or EGFP-SCs suspended in DMEM-F12 media (6 μL) was injected. Injections were performed using a 10 μL siliconized Hamilton syringe with a pulled, beveled glass pipette tip (120 μm diameter), held in a micromanipulator with a micro-injector at a flow rate of 2 μL/min (World Precision Instruments Ltd., Sarasota, FL, USA) as described earlier [55]. The pipette was kept in place for an additional period of 3 min following the injection to minimize leakage of cells upon withdrawal. SCI only controls received exposure and no injection. Following surgery, the muscle layers and the skin were closed and sutured separately.

### 4.8. Post-Operative Care

The rats were allowed to recover in a warmed cage with easy access to water and food. Gentamicin (5 mg/kg, intramuscular; Abbott Laboratories, North Chicago, IL, USA) was administered immediately post-surgery and then daily for seven days. The analgesic Buprenex (0.03 mg/kg, subcutaneous; Reckitt Benckiser, Richmond, VA, USA) was delivered post-surgery and then daily for two days. Lactated Ringers (5 cc, subcutaneous) was given twice a day for seven days or longer if needed. Bladders were manually expressed by gentle abdominopelvic compression twice daily (Crede method) until bladder function returned. Animals had access to food and water ad libitum [55]. They were caged in pairs with Alpha Dri^®^ bedding (changed three times a week, Fort Worth, TX, USA) and were provided water bottles with long curved sipper tubes for easy access.

### 4.9. Flow Cytometry

A cohort from each treatment group (*n* = 5 animals from the SCI-only and SCI with unlabeled SC transplantation groups) was euthanized using CO_2_ followed by thoracotomy and then transcardially perfused with 300 mL cold saline. A 1 cm piece of the spinal cord at the injury epicenter, encompassing the entirety of the lesion and/or SC graft, was dissected into 5 mL of Hanks’ Balanced Salt solution (HBSS) medium (Invitrogen, Waltham, MA, USA). Samples were first subjected to mechanical dissociation using fine scissors and then enzymatic dissociation with trypsin (2.5 mg, Trypsin-EDTA, Sigma-Aldrich, St. Louis, MO, USA) and collagenase (5 mg) in 5 mL DMEM for 15 min at 37 °C. Dissociated tissue was triturated using a 10 mL Pasteur pipette and the tissue homogenates obtained then passed through a 40-micron cell strainer. Samples were centrifuged for 5 min at 1000 rpm and the resulting cell pellet re-suspended in 5 mL of HBSS. The cell suspension was overlaid on an OptiPrep gradient solution to remove lipid/myelin debris as described previously [59]. Cells were then washed once with HBSS followed by immunostaining using specific fluorescently conjugated antibodies for multicolor flow cytometry. For staining, non-specific antibody binding was first blocked with an anti-CD16/32 solution (FcR block, BD Pharmingen), prior to the addition of selected antibodies for cell surface markers at specific concentrations for incubation on ice for 1h. Cells were then washed once with flow cytometry staining buffer (R&D System), fixed, and permeabilized (BD Cytofix/Cytoperm, BD Bioscience) for 20 min to permit immunostaining with selected antibodies against intracellular proteins for 1 h on ice. Control cell suspensions (unstained and single-color control) were employed for setting compensation. The gating strategy used for the selection of infiltrating macrophage and resident microglial populations was based upon the forward (size) and the side scatter (granularity) of these innate immune cells to eliminate any dead cells or debris in combination with the expression of markers, CD11b-conjugated to Pacific Blue or CD68-conjugated to Phycoerythrin, routinely used to specifically identify macrophages and microglia [60]. To further resolve the phenotype of these CD11b or CD68 innate immune cell populations, additional antibodies (iNOS-FITC and CD38-APC or CD163-FITC and Arg1-APC) were employed and percentages of CD11b or CD68 cells expressing multiple markers following quadrant analysis were calculated and averaged across animals. Similarly, the subset of CD11b innate immune cells expressing pro-inflammatory (TNF-α and IL-1β) and anti-inflammatory (IL-4 and IL-10) cytokines as percentages was also measured using an analogous approach by combination with specific fluorophore-conjugated antibodies. Data acquisition was performed using a LSR-Fortessa-HTS analyzer (BD Bioscience) and analyzed using FACSDiva software Version 6.1.3 (BD Bioscience).

### 4.10. Histology and Immunocytochemistry

At 2 weeks post-SCI, 1 week after SC transplantation, a cohort from each treatment group (*n* = 5 animals from the SCI-only and SCI with EGFP-SC transplantation groups) was deeply anesthetized (100 mg/kg ketamine, 10 mg/kg xylazine), 300 U heparin was injected into the spleen, and the animals were transcardially perfused first with 300 mL of cold saline followed with 500 mL of phosphate-buffered 4% paraformaldehyde (0.1 M, pH 7.4; [61]). The T7–T11 thoracic spinal cord (2 cm long piece) that contained the complete lesion and the transplanted SCs was dissected, post-fixed in 4% paraformaldehyde for 48 h, and placed in 30% *w*/*v* sucrose for an additional 48–72 h for cryoprotection during histological processing. A 2 cm piece of the spinal cord encompassing the T7-T9 thoracic cord was cryosectioned into 10 series at 20 μm thickness (each section was separated by a 200 μm interval) for immunohistochemistry and stereological quantification of immune cell numbers and morphology as previously described [62]. Fluorescent-conjugated specific secondary antibodies (ThermoFisher Scientific) were used for visualization by fluorescent microscopy. Slides were cover slipped with Vectashield mounting medium (Vector Labs, Burlingame, CA, USA) and stored at 4 °C until imaged.

### 4.11. Image Analysis

Images of stained tissue sections were acquired using a confocal laser-scanning microscope (Olympus, Fluoview FV 1000, Center Valley, PA, USA). Three to four randomly selected fields were imaged in the region of interest within the tissue section. The immunofluorescence intensity measurements for specific markers on the imaged sections was quantified using Image J software (available online: http://imagej.nih.gov/ij/). For presented images, the tonal range and sharpness (smart sharpen, 0.9 pixels) of the Tiff files were normalized using Adobe Photoshop CS2 (Adobe Systems Inc., San Jose, CA, USA).

### 4.12. Statistical Analysis

The numbers used for the different experimental outcomes were determined by employing a power analysis with SigmaStat (software version 4.0, San Jose, CA, USA) using experimental data obtained previously of a similar type. A power of 0.85 was employed for sample size estimates, meaning that with the *n* chosen, there would be an 85% chance of detecting meaningful differences. Statistical analysis was performed using Graphpad Version 4.0 (Graphpad Software, Inc., La Jolla, CA, USA). A Student’s *t*-test or column statistics was used for comparisons between SC-transplanted and control groups. Differences were accepted as statistically significant at * *p* < 0.05, or ** *p* < 0.01. All errors are given as standard errors of the mean.

## References

[1] Alexander M.S., Anderson K.D., Biering-Sorensen F., Blight A.R., Brannon R., Bryce T.N., Creasey G., Catz A., Curt A., Donovan W. (2009). Outcome measures in spinal cord injury: Recent assessments and recommendations for future directions. Spinal Cord.

[2] Pineau I., Lacroix S. (2007). Proinflammatory cytokine synthesis in the injured mouse spinal cord: Multiphasic expression pattern and identification of the cell types involved. J. Comp. Neurol..

[3] Popovich P.G., Stuckman S., Gienapp I.E., Whitacre C.C. (2001). Alterations in immune cell phenotype and function after experimental spinal cord injury. J. Neurotrauma.

[4] David S., Kroner A., Greenhalgh A.D., Zarruk J.G., Lopez-Vales R. (2018). Myeloid cell responses after spinal cord injury. J. Neuroimmunol..

[5] Ghosh M., Xu Y., Pearse D.D. (2016). Cyclic AMP is a key regulator of M1 to M2a phenotypic conversion of microglia in the presence of Th2 cytokines. J. Neuroinflammation.

[6] Fehlings M.G., Wilson J.R., Harrop J.S., Kwon B.K., Tetreault L.A., Arnold P.M., Singh J.M., Hawryluk G., Dettori J.R. (2017). Efficacy and Safety of Methylprednisolone Sodium Succinate in Acute Spinal Cord Injury: A Systematic Review. Glob. Spine J..

[7] Gensel J.C., Kopper T.J., Zhang B., Orr M.B., Bailey W.M. (2017). Predictive screening of M1 and M2 macrophages reveals the immunomodulatory effectiveness of post spinal cord injury azithromycin treatment. Sci. Rep..

[8] Kobayashi K., Imagama S., Ohgomori T., Hirano K., Uchimura K., Sakamoto K., Hirakawa A., Takeuchi H., Suzumura A., Ishiguro N. (2013). Minocycline selectively inhibits M1 polarization of microglia. Cell Death Dis..

[9] Schaal S.M., Garg M.S., Ghosh M., Lovera L., Lopez M., Patel M., Louro J., Patel S., Tuesta L., Chan W.M. (2012). The therapeutic profile of rolipram, PDE target and mechanism of action as a neuroprotectant following spinal cord injury. PLoS ONE.

[10] Zanier E.R., Pischiutta F., Riganti L., Marchesi F., Turola E., Fumagalli S., Perego C., Parotto E., Vinci P., Veglianese P. (2014). Bone marrow mesenchymal stromal cells drive protective M2 microglia polarization after brain trauma. Neurotherapeutics.

[11] Luz-Crawford P., Jorgensen C., Djouad F. (2017). Mesenchymal stem cells direct the immunological fate of macrophages. Results and Problems in Cell Differentiation.

[12] Nakajima H., Uchida K., Guerrero A.R., Watanabe S., Sugita D., Takeura N., Yoshida A., Long G., Wright K.T., Johnson W.E. (2012). Transplantation of mesenchymal stem cells promotes an alternative pathway of macrophage activation and functional recovery after spinal cord injury. J. Neurotrauma.

[13] Mosher K.I., Andres R.H., Fukuhara T., Bieri G., Hasegawa-Moriyama M., He Y., Guzman R., Wyss-Coray T. (2012). Neural progenitor cells regulate microglia functions and activity. Nat. Neurosci..

[14] DePaul M.A., Palmer M., Lang B.T., Cutrone R., Tran A.P., Madalena K.M., Bogaerts A., Hamilton J.A., Deans R.J., Mays R.W. (2015). Intravenous multipotent adult progenitor cell treatment decreases inflammation leading to functional recovery following spinal cord injury. Sci. Rep..

[15] Gao J., Grill R.J., Dunn T.J., Bedi S., Labastida J.A., Hetz R.A., Xue H., Thonhoff J.R., DeWitt D.S., Prough D.S. (2016). Human neural stem cell transplantation-mediated alteration of microglial/macrophage phenotypes after traumatic brain injury. Cell Transplant..

[16] Bastidas J., Athauda G., De La Cruz G., Chan W.M., Golshani R., Berrocal Y., Henao M., Lalwani A., Mannoji C., Assi M. (2017). Human Schwann cells exhibit long-term cell survival, are not tumorigenic and promote repair when transplanted into the contused spinal cord. Glia.

[17] Kanno H., Pearse D.D., Ozawa H., Itoi E., Bunge M.B. (2015). Schwann cell transplantation for spinal cord injury repair: Its significant therapeutic potential and prospectus. Rev. Neurosci..

[18] Hosseini M., Yousefifard M., Baikpour M., Rahimi-Movaghar V., Nasirinezhad F., Younesian S., Safari S., Ghelichkhani P., Moghadas Jafari A. (2016). The efficacy of Schwann cell transplantation on motor function recovery after spinal cord injuries in animal models: A systematic review and meta-analysis. J. Chem. Neuroanat..

[19] Pearse D.D., Pereira F.C., Marcillo A.E., Bates M.L., Berrocal Y.A., Filbin M.T., Bunge M.B. (2004). cAMP and Schwann cells promote axonal growth and functional recovery after spinal cord injury. Nat. Med..

[20] Anderson K.D., Guest J.D., Dietrich W.D., Bartlett Bunge M., Curiel R., Dididze M., Green B.A., Khan A., Pearse D.D., Saraf-Lavi E. (2017). Safety of autologous human schwann cell transplantation in subacute thoracic spinal cord injury. J. Neurotrauma.

[21] Perry V.H., Brown M.C. (1992). Role of macrophages in peripheral nerve degeneration and repair. Bioessays.

[22] Reichert F., Saada A., Rotshenker S. (1994). Peripheral nerve injury induces Schwann cells to express two macrophage phenotypes: Phagocytosis and the galactose-specific lectin MAC-2. J. Neurosci..

[23] Stratton J.A., Shah P.T. (2016). Macrophage polarization in nerve injury: Do Schwann cells play a role?. Neural Regen. Res..

[24] Mietto B.S., Mostacada K., Martinez A.M. (2015). Neurotrauma and inflammation: CNS and PNS responses. Mediat. Inflamm..

[25] Mokarram N., Merchant A., Mukhatyar V., Patel G., Bellamkonda R.V. (2012). Effect of modulating macrophage phenotype on peripheral nerve repair. Biomaterials.

[26] Martini R., Fischer S., Lopez-Vales R., David S. (2008). Interactions between Schwann cells and macrophages in injury and inherited demyelinating disease. Glia.

[27] Cattin A.L., Burden J.J., Van Emmenis L., Mackenzie F.E., Hoving J.J., Garcia Calavia N., Guo Y., McLaughlin M., Rosenberg L.H., Quereda V. (2015). Macrophage-induced blood vessels guide schwann cell-mediated regeneration of peripheral nerves. Cell.

[28] Tofaris G.K., Patterson P.H., Jessen K.R., Mirsky R. (2002). Denervated schwann cells attract macrophages by secretion of leukemia inhibitory factor (LIF) and monocyte chemoattractant protein-1 in a process regulated by interleukin-6 and LIF. J. Neurosci..

[29] Gaudet A.D., Popovich P.G., Ramer M.S. (2011). Wallerian degeneration: Gaining perspective on inflammatory events after peripheral nerve injury. J. Neuroinflammation.

[30] Damoiseaux J.G., Dopp E.A., Calame W., Chao D., MacPherson G.G., Dijkstra C.D. (1994). Rat macrophage lysosomal membrane antigen recognized by monoclonal antibody ED1. Immunology.

[31] Moriarty L.J., Duerstock B.S., Bajaj C.L., Lin K., Borgens R.B. (1998). Two- and three-dimensional computer graphic evaluation of the subacute spinal cord injury. J. Neurol. Sci..

[32] Leskovar A., Moriarty L.J., Turek J.J., Schoenlein I.A., Borgens R.B. (2000). The macrophage in acute neural injury: Changes in cell numbers over time and levels of cytokine production in mammalian central and peripheral nervous systems. J. Exp. Biol..

[33] Lisi L., Ciotti G.M., Braun D., Kalinin S., Curro D., Dello Russo C., Coli A., Mangiola A., Anile C., Feinstein D.L. (2017). Expression of iNOS, CD163 and ARG-1 taken as M1 and M2 markers of microglial polarization in human glioblastoma and the surrounding normal parenchyma. Neurosci. Lett..

[34] Jablonski K.A., Amici S.A., Webb L.M., Ruiz-Rosado Jde D., Popovich P.G., Partida-Sanchez S., Guerau-de-Arellano M. (2015). Novel markers to delineate murine M1 and M2 macrophages. PLoS ONE.

[35] Amici S.A., Young N.A., Narvaez-Miranda J., Jablonski K.A., Arcos J., Rosas L., Papenfuss T.L., Torrelles J.B., Jarjour W.N., Guerau-de-Arellano M. (2018). CD38 is robustly induced in human macrophages and monocytes in inflammatory conditions. Front. Immunol..

[36] Mitchell D.M., Lovel A.G., Stenkamp D.L. (2018). Dynamic changes in microglial and macrophage characteristics during degeneration and regeneration of the zebrafish retina. J. Neuroinflamm..

[37] Tam W.Y., Ma C.H. (2014). Bipolar/rod-shaped microglia are proliferating microglia with distinct M1/M2 phenotypes. Sci. Rep..

[38] Kigerl K.A., Gensel J.C., Ankeny D.P., Alexander J.K., Donnelly D.J., Popovich P.G. (2009). Identification of two distinct macrophage subsets with divergent effects causing either neurotoxicity or regeneration in the injured mouse spinal cord. J. Neurosci..

[39] Gensel J.C., Zhang B. (2015). Macrophage activation and its role in repair and pathology after spinal cord injury. Brain Res..

[40] Beck K.D., Nguyen H.X., Galvan M.D., Salazar D.L., Woodruff T.M., Anderson A.J. (2010). Quantitative analysis of cellular inflammation after traumatic spinal cord injury: Evidence for a multiphasic inflammatory response in the acute to chronic environment. Brain.

[41] Allison D.J., Ditor D.S. (2015). Immune dysfunction and chronic inflammation following spinal cord injury. Spinal Cord.

[42] Cerqueira S.R., Lee Y.S., Cornelison R.C., Mertz M.W., Wachs R.A., Schmidt C.E., Bunge M.B. (2018). Decellularized peripheral nerve supports Schwann cell transplants and axon growth following spinal cord injury. Biomaterials.

[43] Osaka M., Honmou O., Murakami T., Nonaka T., Houkin K., Hamada H., Kocsis J.D. (2010). Intravenous administration of mesenchymal stem cells derived from bone marrow after contusive spinal cord injury improves functional outcome. Brain Res..

[44] White S.V., Czisch C.E., Han M.H., Plant C.D., Harvey A.R., Plant G.W. (2016). Intravenous transplantation of mesenchymal progenitors distribute solely to the lungs and improve outcomes in cervical spinal cord injury. Stem Cells.

[45] Cho D.I., Kim M.R., Jeong H.Y., Jeong H.C., Jeong M.H., Yoon S.H., Kim Y.S., Ahn Y. (2014). Mesenchymal stem cells reciprocally regulate the M1/M2 balance in mouse bone marrow-derived macrophages. Exp. Mol. Med..

[46] Zheng G., Ge M., Qiu G., Shu Q., Xu J. (2015). Mesenchymal Stromal Cells Affect Disease Outcomes via Macrophage Polarization. Stem Cells Int..

[47] Badner A., Vawda R., Laliberte A., Hong J., Mikhail M., Jose A., Dragas R., Fehlings M. (2016). Early intravenous delivery of human brain stromal cells modulates systemic inflammation and leads to vasoprotection in traumatic spinal cord injury. Stem Cells Transl. Med..

[48] Jessen K.R., Mirsky R. (2016). The repair Schwann cell and its function in regenerating nerves. J. Physiol..

[49] Chen Z.L., Yu W.M., Strickland S. (2007). Peripheral regeneration. Annu. Rev. Neurosci..

[50] Bunge M.B., Bunge R.P., Kleitman N., Dean A.C. (1989). Role of peripheral nerve extracellular matrix in Schwann cell function and in neurite regeneration. Dev. Neurosci..

[51] Stoll G., Griffin J.W., Li C.Y., Trapp B.D. (1989). Wallerian degeneration in the peripheral nervous system: Participation of both Schwann cells and macrophages in myelin degradation. J. Neurocytol..

[52] Pearse D.D., Sanchez A.R., Pereira F.C., Andrade C.M., Puzis R., Pressman Y., Golden K., Kitay B.M., Blits B., Wood P.M. (2007). Transplantation of Schwann cells and/or olfactory ensheathing glia into the contused spinal cord: Survival, migration, axon association, and functional recovery. Glia.

[53] Zhang J., Chen H., Duan Z., Chen K., Liu Z., Zhang L., Yao D., Li B. (2017). The effects of co-transplantation of olfactory ensheathing cells and schwann cells on local inflammation environment in the contused spinal cord of rats. Mol. Neurobiol..

[54] Karperien A., Ahammer H., Jelinek H.F. (2013). Quantitating the subtleties of microglial morphology with fractal analysis. Front. Cell. Neurosci..

[55] Ghosh M., Tuesta L.M., Puentes R., Patel S., Melendez K., El Maarouf A., Rutishauser U., Pearse D.D. (2012). Extensive cell migration, axon regeneration, and improved function with polysialic acid-modified Schwann cells after spinal cord injury. Glia.

[56] Morrissey T.K., Kleitman N., Bunge R.P. (1991). Isolation and functional characterization of Schwann cells derived from adult peripheral nerve. J. Neurosci..

[57] Gruner J.A. (1992). A monitored contusion model of spinal cord injury in the rat. J. Neurotrauma.

[58] Patel V., Joseph G., Patel A., Patel S., Bustin D., Mawson D., Tuesta L.M., Puentes R., Ghosh M., Pearse D.D. (2010). Suspension matrices for improved Schwann-cell survival after implantation into the injured rat spinal cord. J. Neurotrauma.

[59] Nguyen H.X., Beck K.D., Anderson A.J. (2011). Quantitative assessment of immune cells in the injured spinal cord tissue by flow cytometry: A novel use for a cell purification method. J. Vis. Exp..

[60] Martin E., El-Behi M., Fontaine B., Delarasse C. (2017). Analysis of microglia and monocyte-derived macrophages from the central nervous system by flow cytometry. J. Vis. Exp..

[61] Lo T.P., Cho K.S., Garg M.S., Lynch M.P., Marcillo A.E., Koivisto D.L., Stagg M., Abril R.M., Patel S., Dietrich W.D. (2009). Systemic hypothermia improves histological and functional outcome after cervical spinal cord contusion in rats. J. Comp. Neurol..

[62] Barakat D.J., Gaglani S.M., Neravetla S.R., Sanchez A.R., Andrade C.M., Pressman Y., Puzis R., Garg M.S., Bunge M.B., Pearse D.D. (2005). Survival, integration, and axon growth support of glia transplanted into the chronically contused spinal cord. Cell Transplant..

